# Electrophysiological and Behavioral Responses of an Ambrosia Beetle to Volatiles of its Nutritional Fungal Symbiont

**DOI:** 10.1007/s10886-021-01263-0

**Published:** 2021-03-24

**Authors:** Christopher M. Ranger, Marek Dzurenko, Jenny Barnett, Ruchika Geedi, Louela Castrillo, Matthew Ethington, Matthew Ginzel, Karla Addesso, Michael E. Reding

**Affiliations:** 1 Horticultural Insects Research Laboratory, USDA-Agricultural Research Service, 1680 Madison Ave, Wooster, OH 44691 USA; 2 Slovak Academy of Sciences, Institute of Forest Ecology, L’udovíta Štúra 2, 960 53 Zvolen, Slovakia; 3 Present Address: Department of Integrated Forest and Landscape Protection, Technical University in Zvolen, Ul. T. G. Masaryka 24, 960 01 Zvolen, Slovakia; 4 Emerging Pests and Pathogens Research, USDA-Agricultural Research Service, Ithaca, NY 14853-2901 USA; 5 Department of Entomology, Purdue University, 901 W. State Street, West Lafayette, IN 47907 USA; 6 Department of Forestry and Natural Resources, Purdue University, 715 W. State Street, West Lafayette, IN 47907 USA; 7 Otis L. Floyd Nursery Research Center, College of Agriculture, Tennessee State University, McMinnville, TN 37110 USA

**Keywords:** *Xylosandrus germanus*, *Ambrosiella grosmanniae*, Symbiosis, Fungal volatiles

## Abstract

Ambrosia beetles (Coleoptera: Scolytinae) cultivate their fungal symbiont within host substrates as the sole source of nutrition on which the larvae and adults must feed. To investigate a possible role for semiochemicals in this interaction, we characterized electrophysiological and behavioral responses of *Xylosandrus germanus* to volatiles associated with its fungal symbiont *Ambrosiella grosmanniae*. During still-air walking bioassays, *X. germanus* exhibited an arrestment response to volatiles of *A. grosmanniae*, but not antagonistic fungi *Beauveria bassiana*, *Metarhizium brunneum*, *Trichoderma harzianum*, the plant pathogen *Fusarium proliferatum*, or malt extract agar. Solid phase microextraction-gas chromatography-mass spectrometry identified 2-ethyl-1-hexanol, 2-phenylethanol, methyl benzoate and 3-methyl-1-butanol in emissions from *A. grosmanniae*; the latter two compounds were also detected in emissions from *B. bassiana*. Concentration-responses using electroantennography documented weak depolarizations to *A. grosmanniae* fungal volatiles, unlike the comparatively strong response to ethanol. When tested singly in walking bioassays, volatiles identified from *A. grosmanniae* elicited relatively weak arrestment responses, unlike the responses to ethanol. *Xylosandrus germanus* also exhibited weak or no long-range attraction to the fungal volatiles when tested singly during field trials in 2016–2018. None of the fungal volatiles enhanced attraction of *X. germanus* to ethanol when tested singly; in contrast, 2-phenylethanol and 3-methyl-1-butanol consistently reduced attraction to ethanol. Volatiles emitted by *A. grosmanniae* may represent short-range olfactory cues that could aid in distinguishing their nutritional fungal symbiont from other fungi, but these compounds are not likely to be useful as long-range attractants for improving detection or mass trapping tactics.

## Introduction

Ambrosia beetles (Curculionidae: Scolytinae and Platypodinae) are characterized by their obligate symbiosis with fungi (Hulcr et al. 2015). Thirty of the 60 exotic Scolytinae established in North America are within the tribe Xyleborini, which includes many species that attack horticultural trees (Agnello et al. 2017; Gomez et al. 2018; Hulcr and Stelinski 2017; Ranger et al. 2016). Dispersing female xyleborine beetles carry spores of their fungal symbiont(s) within mycetangia in the form of pits, grooves, sacs, or invaginated pouches (Hulcr et al. 2015; Vega and Biedermann 2020). The fungal symbionts are mainly in the genera *Ambrosiella*, *Fusarium*, and *Raffaelea* and rely on ambrosia beetles for dispersal and propagation (Kostovcik et al. 2015; Mayers et al. 2015; Wingfield et al. 2017). During tunnel excavation by female xyleborine beetles into host trees, spores are transferred to the tunnel walls for establishing fungal gardens (Batra 1985). Females begin ovipositing eggs after sowing the fungal symbiont that serves as the sole source of nourishment for developing larvae and maturing adults (Biedermann and Taborsky 2011).

*Xylosandrus germanus* is a xyleborine ambrosia beetle native to southeast Asia but now established throughout much of Europe and North America (Dzurenko et al. 2021; Gomez et al. 2018). Male *X. germanus* are flightless, do not possess a mycetangium, and remain in or near their natal gallery for life; females disperse to initiate new colonies and attack recently cut logs, saplings, and mature trees of more than 200 species in managed and unmanaged systems (Galko et al. 2018; Ranger et al. 2016, 2021). Thin-barked deciduous species are commonly selected in horticultural systems, but coniferous species are also attacked. Despite a broad host range, living but weakened trees in the early stages of physiological stress are preferentially attacked by *X. germanus* while healthy trees are rarely attacked and poorly colonized (Ranger et al. 2015). A variety of factors can predispose trees to attack by *X. germanus*, including flood and low temperature stress (Ranger et al. 2021).

During host-selection, ethanol represents an important long-range attractant and host acceptance cue used by *X. germanus* to locate suitable trees for establishing their nutritional symbiont *Ambrosiella grosmanniae* and rearing offspring (Klimetzek et al. 1986; Ranger et al. 2021; Rassati et al. 2020). Ethanol also promotes the growth of *A. grosmanniae*, and its presence within host tissues increases the colonization success of *X. germanus* (Ranger et al. 2018). Ethanol is induced and emitted from the epidermis of trees in response to the aforementioned stressors and a variety of other abiotic and biotic factors (Kimmerer and Kozlowski 1982; Ranger et al. 2021). Other host-derived compounds tested to date were weak and inconsistent attractants for *X. germanus* when tested alone or in combination with ethanol, including an alcohol (i.e. methanol), aldehyde (i.e. acetaldehyde), ketone (i.e. acetone), spiroacetal (i.e. conophthorin), and several monoterpenes (i.e. α- and β-pinene, camphene, myrcene, ρ-cymene, limonene, and eucalyptol) (Dodds and Miller 2010; Kohnle et al. 1992; Miller et al. 2015; Ranger et al. 2010, 2011, 2014; VanDerLaan and Ginzel 2013). There is currently no evidence that *X. germanus* or other xyleborines produce a long-range aggregation or sex pheromone, perhaps because males are flightless and females reproduce through haplodiploidy (Ott 2007; Ranger et al. 2021).

A growing body of research indicates that insects respond to fungal volatile organic compounds associated with their sensory environment, but few fungal volatiles have been tested to date for activity in laboratory or natural settings (Davis et al. 2013). As fungus farming insects, ambrosia beetles represent a promising model system for symbiosis due to their close association with fungal species. During olfactometer studies, Hulcr et al. (2011) demonstrated that three species of ambrosia beetles were attracted to volatiles emitted from the mycelium of their fungal symbionts, namely, *Xyleborus glabratus* and *Raffaelea lauricola*, *Xyleborus ferrugineus* and *Ambrosiozyma ambrosiae*, and *Xylosandrus crassiusculus* and *Ambrosiella roeperi*. In contrast, the aforementioned species were non-responsive or repelled by mycoparasitic *Trichoderma* sp. Olfactometer studies conducted by Egonyu and Torto (2018) also observed that *Xylosandrus compactus* was attracted to volatiles emitted from mycelium of its symbiont *Fusarium solani* (Mart.) Sacc. Characterizing ambrosia beetle semiochemicals emitted by their fungal symbiont could provide insight into the evolutionary and ecological basis for such chemical signals. A specific and conserved association has been documented among populations of *X. germanus* and *A. grosmanniae* (Mayers et al. 2015), but other ambrosia beetles are associated with multiple different fungi (Kostovcik et al. 2015). Identifying these semiochemicals might also enhance attraction to existing lures for detecting and monitoring destructive ambrosia beetles.

The overall goal of our current study was to characterize the response of *X. germanus* to volatiles associated with its fungal symbiont *A. grosmanniae*. We hypothesized that *X. germanus* would exhibit short- and/or long-range behavioral responses to volatiles associated with *A. grosmanniae* due to their close evolutionary and ecological associations. To test this hypothesis, the specific objectives were to: (1) compare the short-range arrestant response of *X. germanus* to volatiles emitted from *A. grosmanniae* relative to the entomopathogenic fungi *Beauveria bassiana* and *Metarhizium brunneum*, the mycoparasitic fungus *Trichoderma harzianum*, and the plant pathogen *Fusarium proliferatum*; (2) identify volatiles emitted from *A. grosmanniae* and the aforementioned fungi by solid phase microextraction-gas chromatography-mass spectrometry (SPME-GC-MS); (3) characterize olfactory responses of *X. germanus* by means of electroantennography (EAG) to *A. grosmanniae* fungal volatiles; and (4) evaluate the short- and long-range behavioral response of *X. germanus* to *A. grosmanniae* fungal volatiles.

## Methods and Materials

### Culturing of *A. grosmanniae*

Adult female *X. germanus* were collected after dispersing from their overwintering galleries within host tree substrates using bottle traps (Ranger et al. 2010). Traps were baited with an ethanol sachet lure (65 mg/d at 30 °C; AgBio, Inc., Westminster, CO) and deployed in a mixed hardwood forested area at the Ohio Agricultural Research and Development Center in Wayne Co., Ohio, USA (40°45′40.85”N, 81°51′14.71”W). Adults collected in the traps were prevented from desiccating by placing a moistened paper towel rolled into a tube in the bottom collection vessel of the trap (Ranger et al. 2015). Trap contents were collected daily and transferred to parafilm-sealed petri dishes containing moistened filter paper and stored for 24 to 48 h at 3.3 °C. Field collected beetles were then surface sterilized by briefly (2–3 s) dipping in 70% ethanol (aq). Under a laminar flow hood, a sterile transfer needle was next inserted into the thoracic mycetangia of *X. germanus* and streaked onto petri dishes containing 2% malt extract agar. A representative cultured strain isolated from beetles field collected beetles in Shreve, Ohio, USA (40°41′36.24”N; 81°55′31.59”W) in May 2010 and designated as XgOH11 was used (Castrillo et al. 2016; Mayers et al. 2015).

### Behavioral Bioassay

**Fig. 1:**
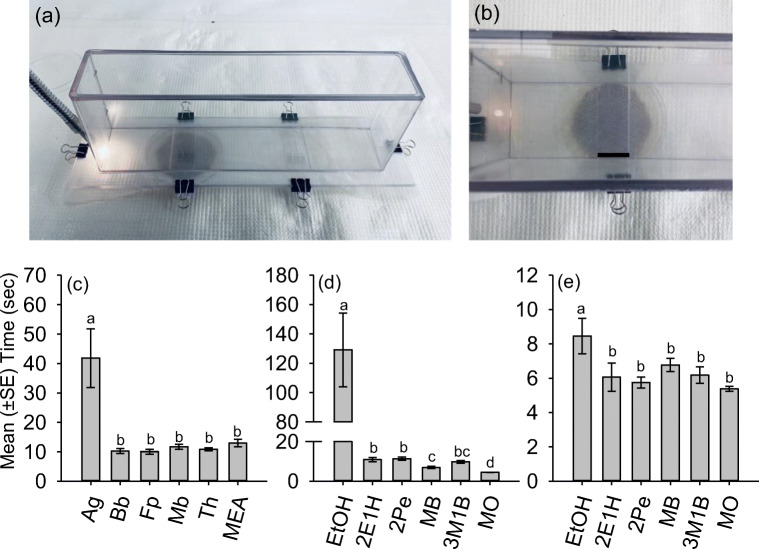
**a-e. a**, **b** Still-air walking olfactometer used to measure the arrestment response of female *X. germanus* to volatile stimuli. As beetles walked along the platform towards the light source, the duration of time was measured that beetles spent within the confines of the mesh-covered rectangular opening above the petri-dish on the left side (indicated by black horizontal bar in 1B, 2.5 cm). **c** Duration of time that *X. germanus* spent above petri dishes containing cultures of *X. germanus*’ nutritional fungal symbiont *Ambrosiella grosmanniae*, *Ag*; entomopathogenic fungi *Beauveria bassiana*, *Bb*, and *Metarhizium brunneum*, *Mb*; mycoparasitic fungus *Trichoderma harzianum*, *Th*; fungal plant pathogen *Fusarium proliferatum*, *Fp*; and malt extract agar, MEA. Duration of time that *X. germanus* spent above petri dishes containing filter paper treated with 40 μl of **d** 1 mg/ml and **e** 0.1 mg/ml dilutions of ethanol, EtOH; 2-ethyl-1-hexanol, 2E1H; 2-phenylethanol, 2PE; methyl benzoate, MB; 3-methyl-1-butanol, 3M1B; and mineral oil, MO. Different letters within a graph represent significantly different means using generalized linear models and least square means ((**c**): χ^2^ = 100.70; df = 5; *P* < 0.0001; (**d**): χ^2^ = 249.64; df = 5; *P* < 0.0001; (**e**): χ^2^ = 17.28; df = 5; *P* = 0.004)

A still-air walking olfactometer (Fig. 1a, b) described by Borden et al. (1968) was used to assess the behavioral responses of *X. germanus* to volatiles of its fungal symbiont, *A. grosmanniae*, as compared to the entomopathogenic fungi *Beauveria bassiana* (Balsamo) Vuillemin strain GHA and *Metarhizium brunneum* Petch strain F52 (=ARSEF 5198; previously identified as *Metarhizium anisopliae*), the mycoparasitic fungus *Trichoderma harzianum* Rifai strain T-22, and the plant pathogen *Fusarium proliferatum* (Matsushima) Nirenberg as representative of a non-symbiont and non-entomopathogen. All fungal strains are maintained under long-term storage at the USDA-ARS Robert W. Holley Center for Agriculture & Health in Ithaca, NY (Castrillo et al. 2016).

The still-air olfactometer consisted of a Plexiglas acrylic platform (36 × 10 cm; L × W) on which ambrosia beetles could walk that included two rectangular openings (5 × 2.5 cm; L × W) positioned 10 cm from either end (Fig. 1a, b). Fine mesh polyester fabric (35 × 35 squares/cm^2^) was secured tightly across the surface of the platform using medium size binder clips (3.17 cm width; 1.6 cm clip capacity) to provide traction for beetles while walking and to allow beetles to pass over the two rectangular openings. The platform was then rested on two uncovered polystyrene petri dishes so the two rectangular openings were positioned directly over the open petri dishes. In doing so, volatiles could emanate upwards from the petri dishes and through the mesh-covered rectangular openings. The petri dish positioned on the right-hand side of the observer was empty and not used for measuring behavioral responses during all bioassays, while the petri dish on the left-hand side of the observer contained a clean MEA plate or a culture of *A. grosmanniae*, *B. bassiana*, *M. brunneum*, *T. harzianum*, or *F. proliferatum*. Cultures were established on the MEA plates and maintained at 20 °C for about 14 d before use in the bioassays. Following SPME-GC-MS analyses, additional bioassays were conducted to assess the arrestment response of *X. germanus* to the following individual compounds: ethanol, 2-ethyl-1-hexanol, 2-phenylethanol, methyl benzoate, 3-methyl-1-butanol, and a mineral oil control. A strip of filter paper (4 × 1 cm; L × W) was treated with 40 μl of 0.1 or 1 mg/ml dilutions in mineral oil of the aforementioned compounds to deliver 4 μg and 40 μg, respectively. After saturation, the filter paper strip was placed in the center of the petri dish underneath the left-hand threshold of the walking platform.

A plexiglass chamber (30 × 6 × 10.5 cm; L × W × H) enclosed on all sides except for the underside was then rested on top of the platform to confine beetles during the walking bioassays. The chamber was removed to place a beetle on the walking platform and then promptly returned. Since recently-emerged ambrosia beetles exhibit a positive phototaxic response (Borden et al. 1968; Ranger pers. obs.), a single gooseneck illuminator (150 W halogen bulb; 1500 lm/m^2^ at 2.54 cm from end of illuminator) generally used for a stereomicroscope was positioned at the end of the walking platform on the left-most side to attract beetles during the walking bioassays. Bioassays were otherwise conducted in a completely dark room.

Assessing the behavioral response of *X. germanus* to volatiles of the aforementioned cultures or individual compounds was initiated by using forceps to place a single adult female near the end of the walking platform opposite the light source. Recently-emerged 3–4 d old laboratory-reared beetles (Castrillo et al. 2012) were held at room temperature in a laboratory exposed to natural light in a petri dish containing moistened filter paper for 3–5 d before use in bioassays. Beetles typically began walking towards the light source within 30 s of being transferred to the platform; beetles that did not exhibit a rapid positive phototaxic response were removed from the chamber and replaced. As beetles walked along the platform towards the light source, a handheld timer was used to record the duration of time that beetles spent within the confines of the mesh-covered rectangular opening in the platform.

Bioassays were conducted during 13:00 to 18:00 EST because *X. germanus* exhibits peak phototaxic behavioral and flight activity during these hours (Ranger, pers. obs.). Clean, fresh mesh fabric was used over the walking platform to test each culture or compound. The plexiglass platform and chamber were wiped clean with warm, unscented soapy water, dried using a paper towel, and allowed to air dry prior to testing each culture or compound. Lab-reared *X. germanus* (*n* = 20 individual beetles per culture or compound) were only used once to assess the behavioral response to *A. grosmanniae*, *B. bassian*a, *M. brunneum*, *T. harzianum*, *F. proliferatum*, and MEA, along with bioassays testing ethanol, 2-ethyl-1-hexanol, 2-phenylethanol, methyl benzoate, 3-methyl-1-butanol, and mineral oil. Individual beetles were not exposed to multiple cultures or compounds. To balance treatment presentation, at least five beetle replicates were measured on a given day using all the cultures or individual volatile compounds (except for *F. proliferatum* since it was tested after the other cultures).

Generalized linear models (PROC GENMOD; SAS Institute Inc., Cary, NC) were used to compare the duration of time *X. germanus* spent within the threshold above the petri dishes containing the various cultures or individual compounds. Due to non-normality, data were initially modeled using the log link function and a Poisson distribution. Goodness of fit for the model and overdispersion was then assessed using the scaled deviance (G^2^/df) parameter. When overdispersion was identified by a large departure >1.0 for the scaled deviance parameter, a negative binomial distribution and log link function was used to fit the model. Differences of least square means were used for pairwise comparisons on treatment effects with significant F-test values from analysis of variance (α = 0.05).

### Analysis of Volatile Emissions

Solid phase microextraction-gas chromatography-mass spectrometry was used to collect and identify volatile emissions from *A. grosmanniae*, *B. bassiana*, *M. brunneum*, *T. harzianum*, *F. proliferatum*, and a MEA control. Briefly, cultures were grown on MEA in glass test tubes (20 × 150 mm) with a threaded black phenolic screw cap. After autoclaving the test tubes and MEA, an aliquot of 6.5 mL of agar was pipetted per test tube. Tubes were then placed in a test tube rack with a 20° tilt to allow the media to solidify at an angle. Test tubes were inoculated about 14 d before using in SPME-GC-MS analyses like the behavioral bioassays.

**Fig. 2:**
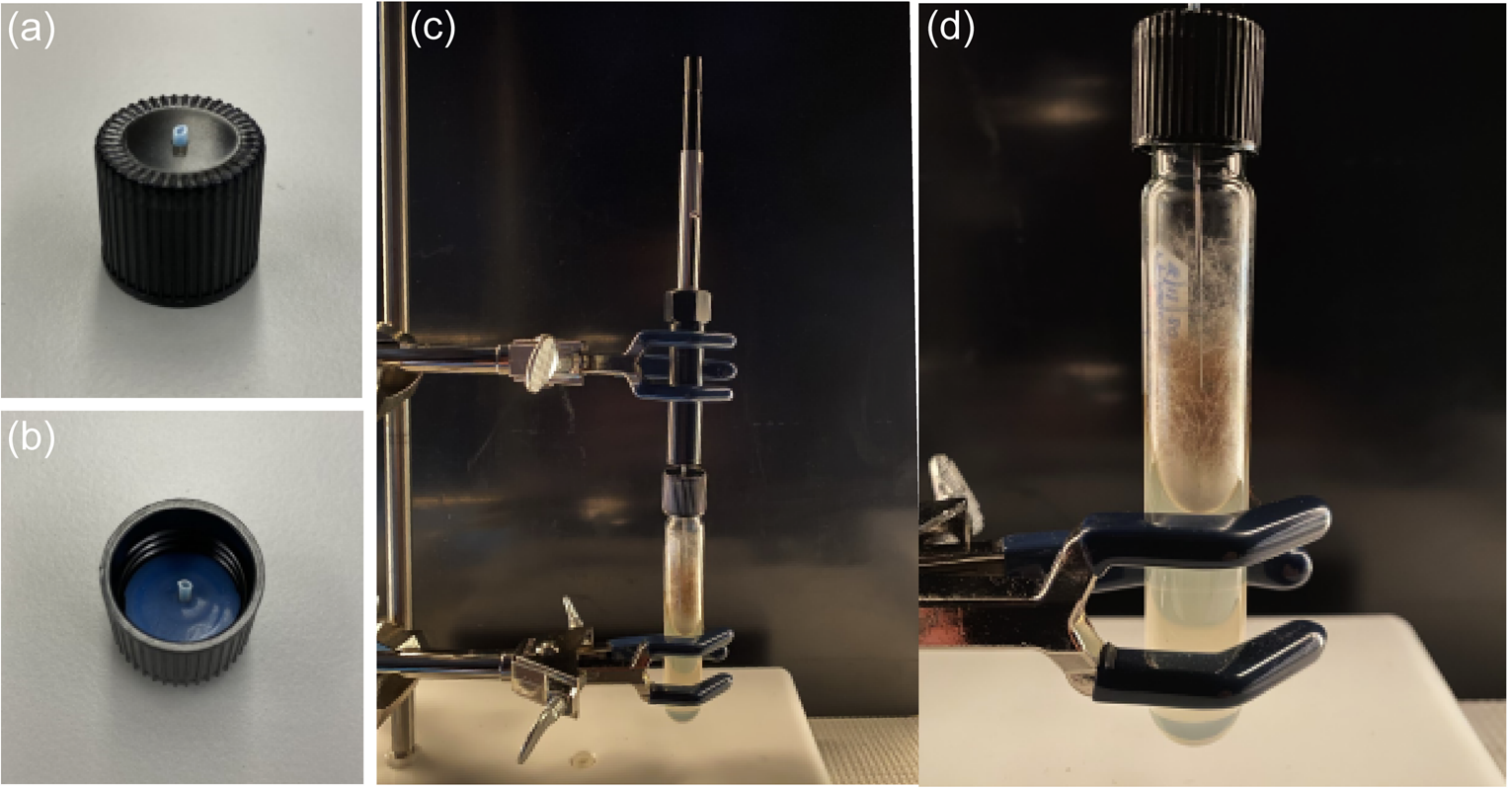
**a-d** Solid phase microextraction (SPME) sampling technique to collect volatiles emitted from mycelium of *A. grosmanniae* (pictured), *B. bassiana*, *M. brunneum*, *T. harzianum*, and *F. proliferatum*. Teflon tubing and septum material were fitted through a pre-drilled hole in the cap (**a, b**) to introduce the syringe into the test tube for fiber exposure, thereby avoiding the possibility of contaminating the SPME syringe and GC-MS inlet liner with silicone septa

For sampling culture volatiles by SPME, a 1 mm diam. Hole was pre-drilled into the screw cap of the test tube (Fig. 2a, b). An autoclaved cork borer was used to cut a circular disk (1.3 cm) from a blue septa silicone sheet (3 mm thick; Chromatography Research Supplies Inc., Louisville, KY). A needle was then used to puncture a hole through the center of the septum disk, and a piece of PTFE microbore tubing (0.81 mm I.D., 1.42 O.D.; Cole-Parmer, Vernon Hills, IL) was pulled through the hole in the septum disk (Fig. 2a). The disk with tubing in place was then inserted into the threaded test tube cap with the Teflon tubing extending out the top of the cap. The purpose of the PTFE tubing was to avoid puncturing the silicone septa and potentially contaminating the GC inlet liner during thermal desorption. Standard caps on the culture tubes were replaced with the modified and autoclaved SPME sampling caps immediately before sampling. A syringe containing a retracted SPME fiber (CAR/PDMS; 75 µm coating; Sigma-Aldrich, St. Louis, Missouri) was then inserted through the test tube cap via the Teflon tubing and secured using a metal clamp and retort stand to position the end of the exposed fiber about 1 cm above the cultures (Fig. 2b). Four tubes with cultures incubated for about 14 d after inoculating were analyzed for each species, along with MEA controls. Sampling was conducted on a laboratory benchtop with an ambient temperature of 21 °C and overhead fluorescent lighting. Fibers were exposed for 30 min and then immediately analyzed by GC-MS.

Fibers were thermally desorbed for 2 min at 225 °C in the injection port of an Agilent 7890B GC (Agilent Technologies, Palo Alto, California) with a SPME liner (0.75 mm × 6.35 mm × 78.5 mm, i.d. × o.d. × length; Restek, Bellefonte, Pennsylvania) under splitless mode with 2 min splitter off time. A DB-5MS column (0.25 µm × 30 m × 0.25 mm; i.d. × length × film thickness; cross-linked/surface bonded 5% phenyl, 95% methylpolysiloxane; Agilent J&W, Santa Clara, California) and a temperature program of 50–250 °C at 3 °C/min were used for the analyses. An Agilent 5977A mass spectrometer was operated in electron impact mode with a scan range of 40–415 amu. Compounds that were unique to the aforementioned fungal cultures and absent in MEA volatile emissions were tentatively identified using NIST library searches. The following identifications were confirmed by comparing retention times and fragmentation patterns with authentic standards (Sigma-Aldrich): 3-methyl-1-butanol (≥99.0% chemical purity), 2-methyl-1-butanol (≥98.0% purity), 2-ethyl-1-hexanol (≥99.0% purity), methyl benzoate (≥99.5% purity), 2-phenylethanol (≥99.0% purity), 1-octen-3-ol (≥98.0% purity), 2-octenal (≥95.0% purity), methyl cinnamate (≥99.0% purity), and α-cedrene (≥95.0% purity).

### Olfactory Responses

Electroantennography (EAG) was used to measure antennal olfactory responses of 5 d old laboratory-reared female *X. germanus* to 2-ethyl-1-hexanol, 2-phenylethanol, methyl benzoate, 3-methyl-1-butanol, and ethanol. As described in greater detail by Ranger et al. (2014), the recording and indifferent electrodes contained Beadle-Ephrussi saline and a silver wire held in place using stainless steel electrode holders (Syntech, Buchenbach, Germany). The indifferent electrode was inserted into the foramen of a recently-excised *X. germanus* head, and the recording electrode was directed to the center of the antennal club where the majority of porous olfactory sensilla are located (Ranger et al. 2017). Micromanipulators were magnetically mounted onto the surface of an antivibration table and recordings were made within a Faraday cage (CleanBench, TMC, Peabody, MA). Antennal preparations were positioned at the end of a stainless steel odor delivery tube (diameter 0.64 cm) through which humidified and carbon-filtered air passed at 150 mL/min at approximately 2.5 cm/s.

Dilutions of 2-ethyl-1-hexanol, 2-phenethylethanol, methyl benzoate, 3-methyl-1-butanol, and ethanol (≥99.5; Sigma-Aldrich) were prepared in mineral oil to achieve concentrations of 0.001, 0.01, 0.1, and 1.0 mg/mL. A 20 µL aliquot of an individual dilution was applied to a filter paper strip (2.5 cm × 0.5 cm, l × w) within a disposable glass Pasteur pipette. The pipette tip was inserted into a hole in the odor delivery tube about 10.8 cm upwind of the antennal preparation. Antennae were exposed to a 0.5-s stimulus puff delivered by a Stimulus Controller CS 55 (Syntech) through the Pasteur pipette at 30 mL/min.

Terpinolene (1.0 mg/mL delivered as 20 ​µg in 20 µL; >92% purity, Contech Enterprises, Inc., Victoria, BC) was used as a reference stimulus (Ranger et al. 2014). Delivery of the reference stimulus always preceded and followed stimulus puff dilutions of the aforementioned stimuli at ascending concentrations. A duration of 60 s was allowed to elapse between puffs. Concentration-response to each volatile stimulus was assessed using 16 separate antennal preparations. Signals from the recording electrode were pre-amplified by a high impedance probe (Universal Single Ended Probe, Syntech) and further amplified, filtered and optimized using a two-channel data acquisition controller (IDAC-2; Syntech). The EAG peak amplitude was measured (mV) and then normalized relative to the response to terpinolene using the following calculation: [(response to analyte)/(initial + final response to terpinolene/2)] × 100. Normalizing the EAG response with a chemical standard corrects for time-dependent variability in antennal sensitivity and allows relative EAG responses to be compared with different stimuli with different cohorts of test insects (Niogret et al. 2011; Kendra et al. 2012). Generalized linear models (PROC GENMOD) with a normal distribution were used to analyze relative percent EAG responses of *X. germanus* to the four concentrations of each compound (SAS Institute Inc., Cary, NC). Comparisons were also made among the five different compounds at each of the four concentrations. Differences of least square means were used for pairwise comparisons on treatment effects (α = 0.05).

### Field Behavioral Responses

Trapping experiments were conducted under field conditions in 2016–2018 to test the attraction of *X. germanus* to 2-ethyl-1-hexanol, 2-phenylethanol, methyl benzoate, 3-methyl-1-butanol, and ethanol. Bottle-traps were prepared according to Ranger et al. (2010). In short, two rectangular openings (length 12.5 cm, width 6 cm) were cut into the sides of a 1-L plastic bottle to allow the entrance of ambrosia beetles. The 1-L bottle was inverted and the mouth was connected to a 0.5-L plastic bottle using a Tornado Tube (Steve Spangler Science, Englewood, Colorado). Lures were secured using wire within the top of the 1-L bottle and the 0.5-L bottle was partially filled with 20 mL of ethanol-free propylene glycol antifreeze (PEAK® SIERRA®; Old World Industries, LLC, Northbrook, IL) as a killing and preserving agent.

Two field trapping experiments were conducted per year in 2016–2018. The first field experiment in 2016 consisted of traps being baited singly with heat-sealed pouch-style emitters of the following compounds: 2-ethyl-1-hexanol (2.5 mg/d at 25 °C; Synergy Semiochemicals, Burnaby, BC), 2-phenylethanol (3.0 mg/d at 25 °C; Synergy), methyl benzoate diluted to 20% with acetyl tributyl citrate to slow the release rate (10 mg/d at 25 °C; Synergy), 3-methyl-1-butanol (4.5 mg/d at 25 °C; Synergy), and ethanol (16 mg/d at 30 °C; AgBio Inc., Westminster, CO), or non-baited. The traps were positioned 0.6 m above ground level by attaching the inverted end of the 1-L bottle to a metal rod staked in the ground. Traps were arranged in parallel lines in a randomized complete block design with five complete blocks within a deciduous woodlot at the Ohio Agricultural Research and Development Center, Wooster, Ohio (40°45′40.46”N; 81°51′15.78”W). Five m was maintained between adjacent traps within a block and 15 m between adjacent blocks. Traps were deployed from 28–June–2016 to 18–July–2016. Samples were then returned to the laboratory and identified to species level. The second field experiment conducted in 2016 consisted of an ethanol lure combined in the same trap with an individual fungal volatile lure with the aforementioned release rates. Traps were deployed as previously described and arranged in a randomized complete block design with five complete blocks from 18–July–2016 to 8–August–2016 (*n* = 5 traps per treatment). Traps baited singly were re-tested from 12–April–2017 to 3–May–2017, and combined with an ethanol lure from 3–May–2017 to 31–May–2017, using the aforementioned release rates and methods for 2016.

Two additional field trapping experiments were conducted in 2018 using the same randomized complete block design used in 2016–2017 but with the following reduced release rates: 2-ethyl-1-hexanol (0.6 mg/d; Synergy), 2-phenylethanol (0.8 mg/d; Synergy), methyl benzoate diluted to 20% with acetyl tributyl citrate (2.5 mg/d; Synergy), and 3-methyl-1-butanol (1.0 mg/d; Synergy). Lures were tested singly from 16–May–2018 to 29–May–2018 and combined with ethanol from 29–May–2018 to 26–June–2018.

A generalized linear model (PROC GENMOD) was used to analyze trap count data of *X. germanus* (SAS Institute Inc., Cary, NC). Due to non-normality, data were initially modeled assuming a Poisson distribution with a goodness of fit for the model being assessed using the scaled deviance (G^2^/df) parameter. When overdispersion was detected by a large departure from 1.0 for the scaled deviance parameter, a negative binomial distribution and log link function was used to fit the model. Differences of least square means were used for pairwise comparisons on treatment effects with significant F-test values from analysis of variance (α = 0.05).

## Results

### Behavioral Bioassay

During still-air walking bioassays, *X. germanus* exhibited a stronger arrestant response to volatiles of its fungal symbiont *A. grosmanniae* compared to the entomopathogenic fungi *B. bassiana* and *M. brunneum*, the mycoparasitic fungus *T. harzianum*, the plant pathogen *F. proliferatum*, and a MEA control (Fig. 1c). As *X. germanus* oriented along a walking platform towards a light source (Fig. 1a, b), individual beetles spent a significantly longer duration arrested over a culture of *A. grosmanniae* compared to *B. bassiana*, *M. brunneum*, *T. harzianum*, *F. proliferatum*, and a MEA control (Fig. 1c). No difference was detected in the duration of time *X. germanus* spent positioned over the non-symbiont cultures or the MEA control.

Based on SPME-GC-MS analyses, subsequent walking bioassays were conducted to assess the behavioral response of *X. germanus* to 2-ethyl-1-hexanol, 2-phenylethanol, methyl benzoate, and 3-methyl-1-butanol (Fig. 1d-e). When tested singly, *X. germanus* spent a significantly longer duration of time above a strip of filter paper treated with 40 μl of 1 mg/ml (i.e. 40 μg) or 0.1 mg/ml (i.e. 4 μg) of ethanol compared to the remaining compounds or mineral oil (Fig. 1d-e). At 40 μg, *X. germanus* spent a longer duration of time over filter paper treated with 2-ethyl-1-hexanol and 2-phenylethanol compared to methyl benzoate; the shortest duration of time was spent over filter paper treated with mineral oil (Fig. 1d). At 4 μg, there was no difference in the duration of time *X. germanus* spent over filter paper treated with 2-ethyl-1-hexanol, 2-phenylethanol, methyl benzoate, 3-methyl-1-butanol or mineral oil (Fig. 1e).

### Analysis of Volatile Emissions

SPME-GC-MS analysis identified four compounds present in volatile emissions from *A. grosmanniae* growing on MEA, including three alcohols (i.e., 3-methyl-1-butanol, 2-phenylethanol, and 2-ethyl-1-hexanol) and one ester (i.e., methyl benzoate) (Table 1). These compounds were not detected in the MEA controls.

**Table 1:** SPME-GC-MS analysis of volatiles associated with the ambrosia beetle nutritional fungal symbiont *Ambrosiella grosmanniae* (*A.g.*), entomopathogenic fungus *Beauveria bassiana* (*B.b.*), mycoparasitic fungus *Trichoderma harzianum* (*T.h.*), and the fungal plant pathogen *Fusarium proliferatum* (*F.p.*)

Species	Compound^a^	Formula	Mean (±SE) Composition (%)	
*A.g.*	3-Methyl-1-Butanol^‡^	C_5_H_12_O	93.17	± 0.51
	2-Ethyl-1-Hexanol^‡^	C_8_H_18_O	3.21	± 0.25
	Methyl Benzoate^‡^	C_8_H_8_O_2_	1.90	± 0.20
	2-Phenylethanol^‡^	C_8_H_10_O	1.72	± 0.11
*B.b.*	3-Methyl-1-Butanol^‡^	C_5_H_12_O	13.26	± 4.92
	2-Methyl-1-Butanol^‡^	C_5_H_12_O	27.44	± 7.65
	1-Octen-3-ol^‡^	C_8_H_16_O	19.26	± 1.64
	2-Octenal^‡^	C_8_H_14_O	12.27	± 7.53
	Methyl Benzoate^‡^	C_8_H_8_O_2_	5.97	± 1.83
	Methyl Cinnamate^‡^	C_10_H_10_O_2_	10.62	± 0.84
	β-Elemene^†^	C_15_H_24_	11.18	± 3.55
*F.p.*	Acoradiene-derivative^†^	C_15_H_24_	5.20	± 1.30
	α-Cedrene^‡^	C_15_H_24_	12.53	± 3.45
	β-Cedrene^†^	C_15_H_24_	9.53	± 0.18
	Muurolene-derivative^†^	C_15_H_24_	2.48	± 1.03
	α-Acoradiene^†^	C_15_H_24_	70.26	± 1.67
*T.h.*	1-Octen-3-ol^‡^	C_8_H_16_O	3.56	± 1.08
	α-Copaene^†^	C_15_H_24_	21.10	± 1.37
	β-Cedrene^†^	C_15_H_24_	32.82	± 1.33
	γ-Muurolene^†^	C_15_H_24_	12.32	± 0.65
	(+)-Sativene^†^	C_15_H_24_	2.05	± 0.08
	α-Acoradiene^†^	C_15_H_24_	5.18	± 0.31
	γ-Acoradiene^†^	C_15_H_24_	3.99	± 0.24
	Acorenone-derivative^†^	C_15_H_26_O	10.42	± 1.42
	Acorenone^†^	C_15_H_24_O	8.58	± 3.90

^a^Identifications based on comparing mass spectra in the National Institute of Standards and Technology (NIST) library (†), or comparing with NIST and an authentic standard (‡)

Seven compounds were detected by SPME-GC-MS in emissions from the entomopathogenic fungus *B. bassiana* growing on MEA, including three alcohols (i.e., 2-methyl-1-butanol, 3-methyl-1-butanol,1-octen-3-ol), one aldehydehyde (i.e., 2-octenal), two esters (i.e., methyl benzoate, methyl cinnamate), and a tentatively identified sesquiterpene (i.e., β-elemene) (Table 1). SPME-GC-MS detected nine compounds in emissions from the mycoparasitic fungus *T. harzianum* growing on MEA, including one alcohol (i.e., 1-octen-3-ol) and eight tentatively identified sesquiterpenes (Table 1). No unique volatiles were detected in emissions of the entomopathogenic fungus *M. brunneum* as compared to the MEA control. Five tentatively identified sesquiterpenes were detected in emissions from *F. proliferatum* (Table 1).

A comparison of volatile emissions determined that two compounds were detected in emissions from *A. grosmanniae* and *B. bassiana*, namely, 3-methyl-1-butanol and methyl benzoate. Two of the tentatively identified sesquiterpenes were shared between volatile emissions of *T. harzianum* and *F. proliferatum*, namely, α-acoradiene and β-cedrene.

### Electroantennogram Responses

**Fig. 3:**
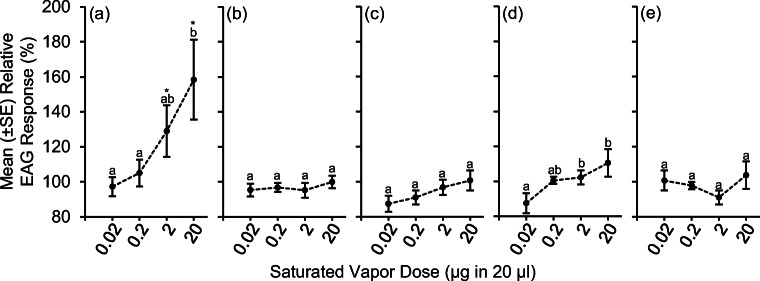
**a-e**. Relative EAG responses of *X. germanus* to serial dilutions of ethanol, EtOH; 2-ethyl-1-hexanol, 2E1H; 2-phenylethanol, 2Pe; methyl benzoate, MB; 3-methyl-1-butanol, 3 MB; and ethanol, EtOH. Relative responses were calculated by normalizing the absolute EAG peak amplitudes relative to the response to saturated vapor concentration of terpinolene (20 μg in 20 μl). Different letters within a graph represent significantly different means using generalized linear models and least square means ((**a**): χ^2^ = 11.40; df = 3; *P* = 0.01; (**b**): χ^2^ = 1.23; df = 3; *P* = 0.75; (**c**): χ^2^ = 4.83; df = 3; *P* = 0.18; (**d**): χ^2^ = 9.33; df = 3; *P* = 0.03; (**e**): χ^2^ = 7.32; df = 3; *P* = 0.06). Asterisks within (**a**) represent a significant difference between saturated concentration of ethanol at 2 μg in 20 μl (χ^2^ = 15.80; df = 4; *P* = 0.0033) and 20 μg in 20 μl (χ^2^ = 18.27; df = 4; *P* = 0.0011) compared to the four other fungal volatiles at these concentrations

Olfactory responses of *X. germanus* were measured using EAG in response to ascending concentrations of 2-ethyl-1-hexanol, 2-phenylethanol, methyl benzoate, 3-methyl-1-butanol, and ethanol (Fig. 3a-e). After normalizing the absolute responses relative to terpinolene, a positive concentration response was documented for ethanol such that 20 μg in 20 μl elicited a significantly larger antennal depolarization response than 0.02 and 0.2 μg in 20 μl (Fig. 3a). A positive concentration response was also documented for methyl benzoate, such that 2 μg and 20 μg in 20 μl elicited significantly larger antennal responses than 0.02 μg in 20 μl (Fig. 3d). A positive antennal concentration response was not observed for 2-ethyl-1-hexanol, 2-phenylethanol, or 3-methyl-1-butanol ranging at concentrations ranging from 0.02–20 μg in 20 μl (Fig. 3b, c, e).

When compared among the five volatile compounds, ethanol at 2 μg and 20 μg in 20 μl elicited significantly larger depolarizations than the remaining compounds at these corresponding concentrations (Fig. 3a). There was no difference in depolarizations for 2-ethyl-1-hexanol, 2-phenylethanol, methyl benzoate, and 3-methyl-1-butanol when tested at concentrations ranging from 0.02–20 μg in 20 μl.

### Field Behavioral Responses

**Fig. 4:**
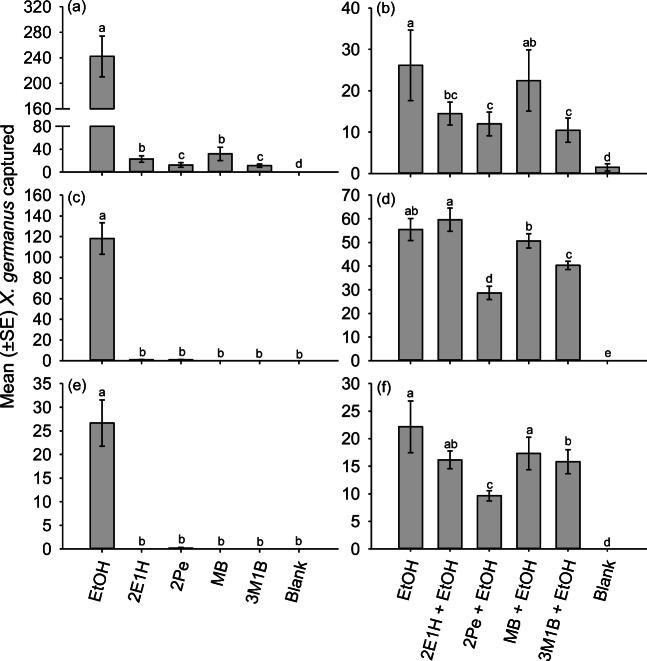
**a-f**. Captures of *X. germanus* in traps deployed in **a**, **b** 2016, **c**, **d** 2017, and **e**, **f** 2018 that were baited singly **a**, **c**, **e** or paired with ethanol **b**, **d**, **f** to test the attractiveness of ethanol, EOH; 2-ethyl-1-hexanol, 2E1H; 2-phenylethanol, 2Pe; methyl benzoate, MB; 3-methyl-1-butanol, 3 MB. The following release rates were tested in 2016–2017: 2-ethyl-1-hexanol (2.5 mg/d), 2-phenylethanol (3.0 mg/d), methyl benzoate (10.0 mg/d), and 3-methyl-1-butanol (4.5 mg/d). The following release rates were tested in 2018: 2-ethyl-1-hexanol (0.6 mg/d), 2-phenylethanol (0.8 mg/d), methyl benzoate (2.5 mg/d), and 3-methyl-1-butanol (1.0 mg/d). Different letters within a graph represent significantly different means using generalized linear models and least square means ((**a**): χ^2^ = 80.83; df = 5; *P* < 0.0001; (**b**): χ^2^ = 36.57; df = 5; *P* < 0.0001; (**c**): χ^2^ = 75.98; df = 5; *P* < 0.0001; (**d**): χ^2^ = 597.16; df = 5; *P* < 0.0001; (**e**): χ^2^ = 48.93; df = 5; *P* < 0.0001; (**f**): χ^2^ = 79.21; df = 5; *P* < 0.0001)

When the fungal volatiles were tested singly at the higher release rate in 2016, significantly more *X. germanus* were attracted to traps baited with ethanol, 2-ethyl-1-hexanol, 2-phenylethanol, methyl benzoate, or 3-methyl-1-butanol compared to the blank control (Fig. 4a). Ethanol alone attracted the most *X. germanus*, while 2-ethyl-1-hexanol and methyl benzoate attracted more *X. germanus* than 2-phenyethanol and 3-methyl-1-butanol (Fig. 4a). When re-tested singly in 2017, there was no difference in the number of *X. germanus* attracted to traps baited with 2-ethyl-1-hexanol, 2-phenylethanol, methyl benzoate, 3-methyl-1-butanol, or the blank control (Fig. 4c). Traps baited with ethanol alone attracted significantly more *X. germanus* than all other compounds.

When paired with ethanol and tested in 2016, traps baited with ethanol plus methyl benzoate attracted a comparable number of *X. germanus* compared to traps baited with ethanol alone (Fig. 4b). In contrast, traps baited with ethanol plus 2-ethyl-1-hexanol, ethanol plus 2-phenylethanol, and ethanol plus 3-methyl-1-butanol attracted fewer *X. germanus* than traps baited with ethanol alone. When re-tested in 2017, traps baited with ethanol plus 2-ethyl-1-hexanol and ethanol plus methyl benzoate attracted a comparable number of *X. germanus* compared to traps baited with ethanol alone (Fig. 4d). In contrast, traps baited with ethanol plus 2-phenylethanol and ethanol plus 3-methyl-1-butanol attracted significantly fewer *X. germanus* than ethanol alone.

In 2018 with reduced rates of the fungal volatiles, there was no difference in the attraction of *X. germanus* to each of the four fungal volatiles tested singly compared to the non-baited blank control (Fig. 4e). When combined with an ethanol lure and tested in 2018, there was no difference in the attraction of *X. germanus* to traps baited with ethanol plus 2-ethyl-1-hexanol and ethanol plus methyl benzoate compared to ethanol alone, whereas 2-phenylethanol and 3-methyl-1-butanol still reduced the attraction of *X. germanus* to ethanol at the lower release rate tested in 2018 (Fig. 4f).

## Discussion

The role of fungal volatiles in mediating interactions among fungi and insect symbionts is receiving increased attention, but has been largely unexplored, especially for bark and ambrosia beetles associated with symbiotic fungi (Cale et al. 2016). As part of our current study, *X. germanus* exhibited a short-range arrestment response to fungal volatiles emitted by the mycelium of its nutritional fungal symbiont, but not to emissions from two entomopathogenic fungi, a mycoparasitic fungus, or a fungal pathogen. SPME-GC-MS identified four volatile compounds in the emissions of *A. grosmanniae* growing on MEA, namely, 2-ethyl-1-hexanol, 2-phenylethanol, methyl benzoate, and 3-methyl-1-butanol. Compared to ethanol, relatively weak antennal depolarizations were exhibited by *X. germanus* to these four fungal volatiles. Weak or no response to the individual volatiles was also documented during walking bioassays and field trials. None of the fungal volatiles enhanced attraction of *X. germanus* to ethanol when tested singly in field trials; in contrast, 2-phenylethanol and 3-methyl-1-butanol consistently reduced attraction to ethanol. Volatiles emitted by *A. grosmanniae* may function as short-range olfactory cues that aid in distinguishing their nutritional fungal symbiont from other fungi, but these compounds do not function as long-range attractants. For instance, olfactory and behavioral responses of *X. germanus* to these compounds could be a function of *A. grosmanniae* being their sole source of nutrition, thereby critical semiochemicals linked to their survival and fitness perhaps by helping to maintain the specific symbiosis between *X. germanus* and *A. grosmanniae*. These responses could also be related to the association of these compounds with host trees (Holighaus and Schütz 2006), or represent compounds often found in the beetles environment (i.e., semiochemical parsimony) as described by Blum (1996). Similarly, the ‘neutral hypothesis’ proposes that microbial volatile emissions might influence insect behavior by coincidence, potentially due to similarity with evolutionarily relevant infochemicals (Davis et al. 2013).

Our current study supports a small body of work demonstrating that ambrosia beetles respond to the volatile profile of their fungal symbiont and can perhaps distinguish it from other fungal volatile profiles. *Xylosandrus compactus* displayed short-range attraction to its fungal symbiont (*F. solani*) during olfactometer bioassays, but comparisons were only made against an agar control (Egonyu and Torto 2018). Similarly, Hulcr et al. (2011) demonstrated that three species of ambrosia beetles exhibited short-range attraction exclusively to fungal volatiles emitted from their symbionts, including *Ambrosiella xylebori*, *Ambrosiozyma* sp., and *Raffaelea lauricola*, but were non-responsive or repelled by *Trichoderma* sp. (Hulcr et al. 2011). However, a limitation of the study by Hulcr et al. (2011) is that a non-pathogenic, non-symbiotic fungal species was not tested. Unlike the response to *A. grosmanniae*, *X. germanus* did not exhibit an arrestment response to volatiles associated with the entomopathogenic fungi *B. bassiana* and *M. brunneum*, the mycoparasitic fungus *T. harzianum*, or the plant pathogen *F. proliferatum* as a representative of a non-pathogenic, non-symbiotic fungus. Additional free-choice studies are warranted to assess what volatiles influence the short-range behavioral response of *X. germanus* to its fungal symbiont. In addition, our SPME-GC-MS analyses tentatively identified a variety of sesquiterpenes from *T. harzianum* that could have repellent activity in support of bioassays by Hulcr et al. (2011).

Ethanol, 2-methyl-1-propanol, and 3-methyl-1-butanol were detected by SPME-GC-MS from an unspecified *Ambrosiella* sp., and ethanol, ethyl acetate, 2-methyl-1-propanol, 3-methyl-1-butanol, and 3-methyl-1-butanol acetate were emitted from *R. lauricola*, the symbiont of *X. glabratus* (Kuhns et al. 2014). SPME-GC-MS analysis of volatile emissions from *F. solani*, the symbiont of *X. compactus*, identified ethanol, 3-methyl-1-butanol, and (*E*)-β-caryophyllene as predominant (Egonyu and Torto 2018). We detected 2-ethyl-1-hexanol, 2-phenylethanol, methyl benzoate, and 3-methyl-1-butanol by SPME-GC-MS from *A. grosmanniae* growing on MEA. SPME can be superior to dynamic headspace sampling for minimizing the loss or obscuration of highly volatile compounds, but vacuum-assisted sampling as part of the latter technique is useful for collecting volatile and semi-volatile compounds (D’Alessandro and Turlings 2006; Jeleń 2003; Morath et al. 2012). For instance, Egonyu and Torto (2018) detected 40 compounds emitted by *F. solani* using dynamic headspace sampling compared to 9 compounds detected by SPME. 3-Methyl-1-butanol was a predominant compound collected using SPME and dynamic headspace sampling from *F. solani* (Egonyu and Torto 2018). Similarly, 3-methyl-1-butanol was a predominant volatile collected by dynamic headspace sampling, along with eight other compounds, from fungal symbionts of the mountain pine beetle (*Dendroctonus ponderosae* Hopkins) (Cale et al. 2016).

Some of the volatiles identified from ambrosia beetle fungal symbionts appear to be ubiquitous, especially 3-methyl-1-butanol (Cale et al. 2016; Egonyu and Torto 2018; Korpi et al. 2009; Kuhns et al. 2014; Morath et al. 2012). Our results suggest *A. grosmanniae* is associated with a relatively simple volatile profile but could have a characteristic odor as documented for other fungi (Morath et al. 2012). Furthermore, volatile blends likely represent more distinguishing information than the presence of individual compounds. Percent compositions associated with SPME analyses in our current study and previous ones (Kuhns et al. 2014) must also be considered qualitatively since calibrations for specific volatile compounds were not performed. Thus, the abundance and ratio of volatiles emitted from *A. grosmanniae* could be quite different from the SPME analyses. As noted by Jeleń (2003), peak areas of individual compounds extracted by SPME can be influenced by a variety of factors, including fiber coatings, temperature, time, pH, and others. The intention of our current study was to establish a qualitative volatile profile of *A. grosmanniae* in pursuit of behaviorally active compounds rather than quantitatively characterize volatile emissions of this fungal symbiont.

Notably, 3-methyl-1-butanol and methyl benzoate were detected in emissions from *A. grosmanniae* and *B. bassiana*, but *X. germanus* did not exhibit an arrestment response to this entomopathogenic fungus during our bioassays. Quantitative analyses of volatile emissions from *A. grosmanniae* and *B. bassiana* would be useful for comparing major, minor, and trace components between these two species. Crespo et al. (2008) detected ethanol, sesquiterpenes, and diisopropyl naphthalenes, while Bojke et al. (2018) detected 3-methylbutanal, fatty acids, and sesquiterpenes by SPME-GC-MS in volatile emissions from *B. bassiana*.

As previously noted, ethanol was detected in emissions from ambrosia beetle fungal symbionts (Egonyu and Torto 2018; Kuhns et al. 2014). We did not detect the emission of ethanol from *A. grosmanniae* at 14 days after inoculating the MEA as part of our current study, but subsequent analyses have detected the emission of ethanol from *A. grosmanniae* at 5 d after inoculating MEA media (Ranger, pers. obs.). The emission of ethanol from vulnerable host trees (Ranger et al. 2021) and by ambrosia beetle fungal symbionts (Egonyu and Torto 2018; Kuhns et al. 2014) could account for the strong behavioral response exhibited by *X. germanus* and other ambrosia beetle to this semiochemical. Since ethanol promotes the growth of *Ambrosiella* spp. and inhibits the growth of fungal garden competitors (Ranger et al. 2018), its emission by *Ambrosiella* spp. might also serve in a defensive capacity to suppress the establishment of antagonists. As the profile of volatiles emitted by fungi can vary depending on substrate, duration of incubation, nutrients, temperature, and other parameters (Morath et al. 2012), additional time-course studies are warranted to further characterize fungal volatiles emitted by *A. grosmanniae.* In particular, volatile profiles should be compared for symbionts growing on MEA alone vs. MEA infused with sawdust from host trees, along with cultures within host tree galleries.

Unlike the concentration-response to ethanol, relatively weak EAG concentration-responses were elicited by 2-ethyl-1-hexanol, 2-phenylethanol, methyl benzoate, and 3-methyl-1-butanol as part of our current study. Due to the close evolutionary and ecological association between ambrosia beetles and their fungal symbionts, the olfactory system of ambrosia beetles could contain narrowly tuned, highly specific olfactory receptors that are wired to dedicated neuronal circuits in response to these ecologically relevant odors (Andersson et al. 2015). Data from our current study does not support this scenario for 2-ethyl-1-hexanol, 2-phenylethanol, methyl benzoate, or 3-methyl-1-butanol, but a narrowly tuned receptor that is highly specific to ethanol could be associated with *X. germanus*. Single sensillum recordings from the antennal club of *X. germanus* would help to characterize the specificity of the different sensilla types, particularly for ethanol (Andersson et al. 2015; Olsson and Hansson 2013).

As compared to ethanol, some arrestment responses were exhibited by *X. germanus* to the individual fungal volatiles tested during the still-air olfactometer bioassays. Still, these results demonstrate the walking bioassay first described by Borden et al. (1968) is useful for measuring the behavioral response of ambrosia beetles. Weak and inconsistent long-range attraction was exhibited by *X. germanus* to the individual fungal volatiles when tested in 2016–2018. None of the compounds enhanced attraction to ethanol, and 2-phenylethanol and 3-methyl-1-butanol consistently reduced attraction to ethanol. 2-Phenylethanol inhibited the response of *Dendroctonus frontalis* Zimmermann and *Dendroctonus ponderosae* Hopkins to attractants (Pureswaran et al. 2000; Sullivan et al. 2007) but enhanced the attraction of *Ips paraconfusus* Lanier to male-infested logs (Renwick et al. 1976). The basis for inhibition could be related to the variety of origins of 2-phenylethanol, including bark beetles and associated microorganisms (Sullivan 2005; Sullivan et al. 2007). Similarly, *X. compactus* exhibited antennal responses to methyl isovalerate and 2,3-butanediol in adsorbent-trapped extracts from *F. solani* during gas chromatography–electroantennography experiments (Egonyu and Torto 2018), but unlike ethanol these compounds were only slightly attractive when tested singly under field conditions.

While semiochemical release rate is critical, Davis et al. (2013) noted that a central question of insect olfactory responses to fungal volatiles is whether the insects perceive the volatiles individually or as a mixture. Individual volatiles are probably not as informative as blends (Davis et al. 2013), as demonstrated for insect responses to plant volatiles (Webster et al. 2008). Still, ethanol alone is highly attractive to ambrosia beetles and emitted from both their host trees (Ranger et al. 2021) and fungal symbionts (Egonyu and Torto 2018; Kuhns et al. 2014). Subsequent studies are being pursued to test blends of volatiles based on the profile emitted from *A. grosmanniae*, but challenges exist with cross-reactivity and release rates among compounds of varying chemical classes when pursuing an optimal lure (Nielsen et al. 2019). Notably, a synthetic blend of fungal volatiles from *R. lauricola* consisting of ethyl acetate: ethanol: isoamyl alcohol: isoamyl acetate (36.5: 29: 22: 12.5) was not alone attractive to *X. glabratus*, but did synergistically enhance attraction to a Manuka oil lure comprised of host volatiles (Kuhns et al. 2014).

Our current findings contribute to a small but growing body of research seeking to characterize how fungal volatiles mediate interactions between ambrosia beetles and their fungal symbionts. Overall, results from our current study indicate that *X. germanus* can sense general volatiles emitted from *A. grosmanniae*, but it is unclear if these compounds are related to symbiosis. Our results also indicate that fungal volatiles identified from *A. grosmanniae* are not promising semiochemicals to enhance the attraction to ethanol for monitoring or mass trapping purposes. Since ethanol represents a strong long-range attractant for *X. germanus* and many other ambrosia beetles, a more effective blend could be difficult to achieve. Still, a range of ecological and practical topics could be addressed, including further characterization of fungal volatiles and factors influencing emissions, olfactory selectivity and sensitivity by ambrosia beetles, and lure optimization.

## Declarations

All the procedures performed in studies involving insects were in accordance with the ethical standards of the institution or practice at which the studies were conducted.

This article does not contain any studies with human participants or vertebrate animals performed by any of the authors.
